# Effects of Electrochemical Hydrogen Charging Parameters on the Mechanical Behaviors of High-Strength Steel

**DOI:** 10.3390/ma17174290

**Published:** 2024-08-30

**Authors:** Wen-Jiao Dan, Hao Shi, Cheng-Wang Tang, Xu-Yang Wang

**Affiliations:** 1 School of Mechanical Engineering, Anhui Science and Technology University, Chuzhou 233100, China; 2 School of Ocean and Civil Engineering, Shanghai Jiao Tong University, Shanghai 200240, China

**Keywords:** structural high-strength steel, electrochemical hydrogen charging, mechanical behaviors, tensile, hydrogen damage

## Abstract

Extended exposure to seawater results in the erosion of the structural high-strength steels utilized in marine equipment, primarily due to the infiltration of hydrogen. Consequently, this erosion leads to a decrease in the mechanical properties of the material. In this investigation, the mechanical responses of Q690 structural high-strength steel specimens were investigated by considering various hydrogen charging parameters, such as the current density, charging duration, and solution concentration values. The findings highlighted the significant impacts of electrochemical hydrogen charging parameters on the mechanical behaviors of Q690 steel samples. Specifically, a linear relationship was observed between the mechanical properties and the hydrogen charging current densities, while the associations with the charging duration and solution concentration were nonlinear. Additionally, the fracture morphology under various hydrogen charging parameters was analyzed and discussed. The results demonstrate that the mechanical properties of the material degrade with increasing hydrogen charging parameters, with tensile strength and yield stress decreasing by approximately 2–4%, and elongation after fracture reducing by about 20%. The findings also reveal that macroscopic fractures exhibit significant necking in uncharged conditions. As hydrogen charging parameters increase, macroscopic necking gradually diminishes, the number of microscopic dimples decreases, and the material ultimately transitions to a fully brittle fracture.

## 1. Introduction

With the progressive development of marine equipment towards increased tonnages, decreased weights, and heightened efficiencies, coupled with the rigorous operational environments they experience, the demands for the strength thresholds of such equipment are escalating. The use of 690 MPa grade high-strength steel in marine equipment has become widespread [1,2]. High-strength steel necessitates cathodic protection measures to prevent corrosion from seawater and the marine atmosphere. However, throughout cathodic protection and corrosion processes, a substantial influx of hydrogen atoms infiltrates the interior of steel, precipitating issues such as cracking, white spot formation, hydrogen-induced damage, and fracture failure [3,4].

Numerous studies have extensively investigated the influences of hydrogen charging on the mechanical properties of steels. Arniella et al. [5] demonstrated that for 2205 duplex stainless steel, the hydrogen embrittlement index increased with increasing hydrogen charging current density and hydrogen content. Wang et al. [6] elucidated that, for high-strength steels, an augmented diffuse hydrogen content corresponded to a decrease in notch tensile strength, with the degree of reduction becoming increasingly pronounced with increasing stress concentration coefficients. Bai et al. [7] examined X80 pipeline steel under varying pre-charging hydrogen time conditions in an H2S environment and reported a decrease in the fracture strain of specimens with a prolonged pre-charging hydrogen time. Liang et al. [8] observed that super duplex stainless steel maintained good austenitic plasticity during tensile tests, while ferritic regions exhibited plasticity loss, with hydrogen embrittlement having a diminished impact on the sample’s center. Dong et al. [9] reported that, for X100 steel, increases in the hydrogen charging time and current density resulted in an increase in hydrogen content and subsequent specimen cracking. Zhao et al. [10] reported severe hydrogen embrittlement damage in Q980 steel after hydrogen charging, accompanied by a transition from tough to brittle fracture behavior. Tian et al. [11,12] noted increased stress corrosion cracking (SCC) sensitivity in E690 steel after hydrogen infiltration, which was attributed to changes in the cathodic potential and hydrogen diffusion behavior. Venezuela et al. [13] demonstrated increased hydrogen embrittlement sensitivity in steel submerged in seawater with increasingly negative cathodic potentials or decreased pH values. Shibayama et al. [14] observed gradual specimen cracking with increasing current density, coupled with a reduced critical hydrogen content under elevated stress levels. Rudomilova et al. [15] characterized the microstructures of CP1000 and DP1000 steel, attributing differences in hydrogen diffusion coefficients to variations in microstructural features and grain sizes. Venezuela et al. [16] investigated the effects of hydrogen on the advanced high-strength steel MS1700 and associated its heightened hydrogen sensitivity with its increased carbon and martensite content, which facilitated enhanced hydrogen trapping. Gong et al. [17] conducted electrochemical hydrogen charging experiments, noting the resultant increases in residual stress and decreases in strength and ductility. Li et al. [18] observed a significant decrease in ductility and the occurrence of intergranular fracture during in situ hydrogen charging tensile tests. Furthermore, the hydrogen charging time decreased the tensile strength and exacerbated the susceptibility to grain boundary hydrogen embrittlement [19,20,21,22]. As the hydrogen charging current density and solution concentration increased, the material’s tensile strength and ductility decreased, culminating in a transition from ductile dimple to intergranular fracture mode and subsequent specimen cracking [23,24,25,26]. Overall, hydrogen decreases the toughness and ductility of high-strength steels, amplifying fracture propensity and weakening material strength. The movement of hydrogen atoms into the steel can induce hydrogen damage and fracture failure, thereby instigating crack formation within the material.

In this investigation, three hydrogen charging types were employed: (1) different hydrogen charging current densities (0.5 mol/L H_2_SO_4_ + 1 g/L H2NCSNH2) for 1 h at current densities of 10 mA/cm^2^, 50 mA/cm^2^, 70 mA/cm^2^, and 90 mA/cm^2^; (2) various hydrogen charging times (0.5 mol/L H_2_SO_4_ + 1 g/L H2NCSNH2) at a current density of 50 mA/cm^2^ for 10 min, 30 min, 1 h, 2 h, and 4 h; and 3) different hydrogen charging solution concentrations (at 50 mA/cm^2^ for 1 h) consisting of 0.5 mol/L H_2_SO_4_ + 1 g/L H2NCSNH2, 0.5 mol/L H_2_SO_4_ + 5 g/L H2NCSNH2, and 0.5 mol/L H_2_SO_4_ + 10 g/L H2NCSNH2. These types were chosen to explore their impacts on the mechanical properties of the Q690 structural high-strength steel. The subsequent analysis was focused on elucidating the variations in the mechanical properties induced by the different hydrogen charging parameters. Finally, the corresponding fracture morphology has been presented and discussed.

## 2. Materials and Experiments

To investigate the mechanical behavior after electrochemical hydrogen charging, Q690 high-strength structural steel was chosen for tensile testing. The main chemical composition of the steel is listed in Table 1, and the mechanical behaviors of the Q690 steel are listed in Table 2.

**Figure 1:**
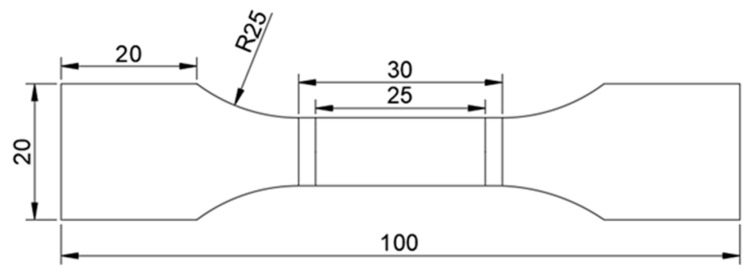
Geometric dimensions of the tensile specimens (mm).

The test specimens were cut from structural steel plates and machined according to the geometric dimensions shown below [27], and the gauge length of the specimen was 25 mm (Figure 1).

**Figure 2:**
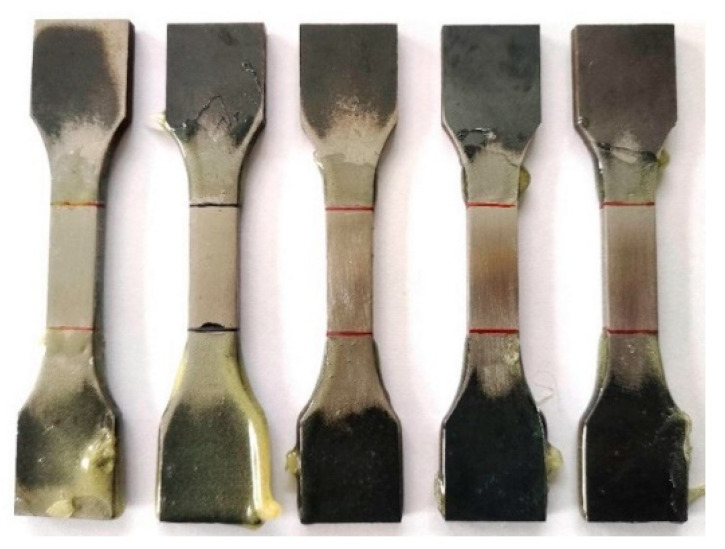
Ground and polished samples before electrochemical hydrogen charging.

Before electrochemical hydrogen charging, the central portion of the test sample underwent grinding and polishing procedures. Initially, a polishing machine was used to grind the middle part of the sample. Subsequently, the samples were subjected to sequential grinding using #400, #800, #1200, and #2000 sandpaper. Following grinding, the sample underwent polishing treatment. Finally, the polished gauge length of the sample was delineated with a red line (the prehydrogenated surface had a length of 25 mm, a width of 10 mm, and a hydrogenated area of 2.5 cm^2^), while epoxy resin was applied to protect the surface of the sample outside the marked area during electrochemical hydrogen charging (Figure 2).

**Figure 3:**
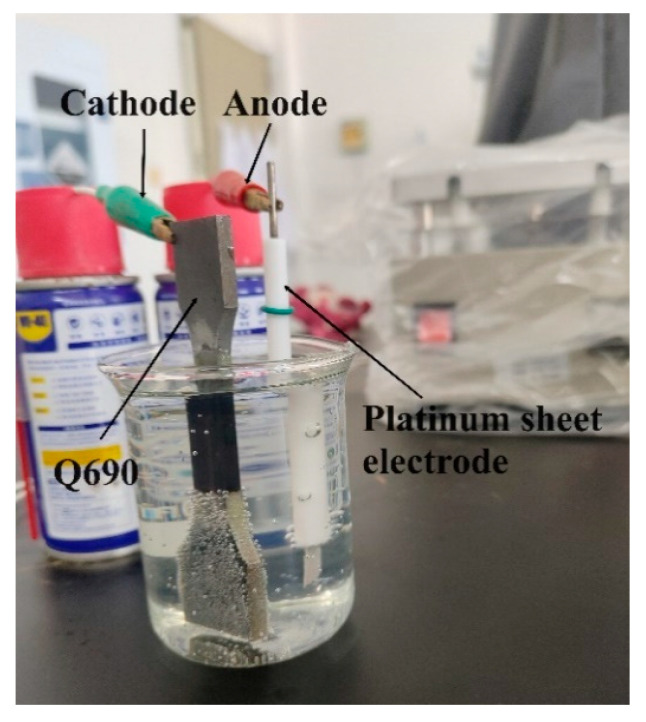
Electrochemical hydrogen charging.

During electrochemical hydrogen charging, the Q690 high-strength steel was connected to the cathode, while a platinum electrode was connected to the anode (Figure 3). Both the cathode and anode were linked to an electrochemical workstation (CHI700E).

Three hydrogen charging parameters were selected for the electrochemical hydrogen charging process (Table 3).

**Figure 4:**
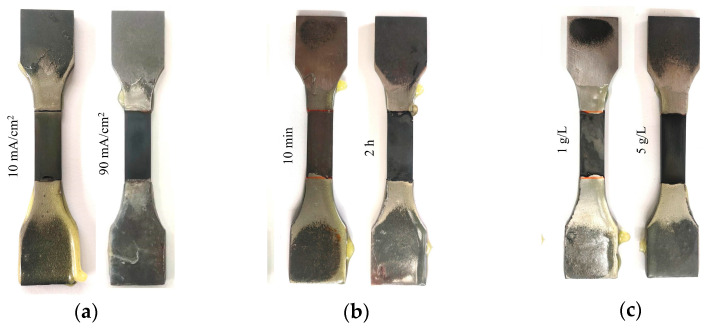
Surfaces of the samples with different hydrogen charging parameters: (**a**) different current densities, (**b**) different times, and (**c**) different solution concentrations.

**Figure 5:**
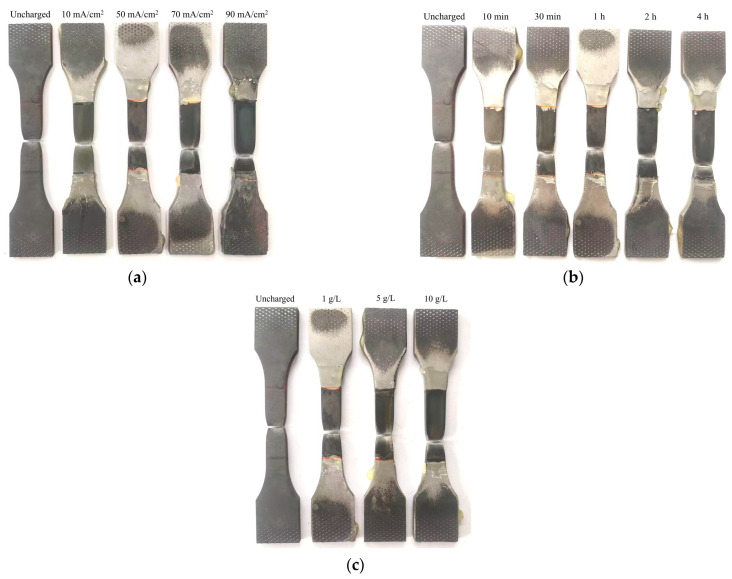
Fracture samples with various hydrogen charging parameters: (**a**) current densities, (**b**) times, and (**c**) solution concentrations.

Three distinct hydrogen charging types were employed in the electrochemical hydrogen charging process, as detailed in Table 3. Type T1 involved applying current densities of 0, 10, 50, 70, and 90 mA/cm^2^ for a duration of 1 h using a solution composed of 0.5 mol/L of H_2_SO_4_ and 1 g/L of H_2_NCSNH_2_. Type T2 employed a constant current density of 50 mA/cm^2^ for varying durations of 1/6, 1/2, 1, 2, and 4 h, utilizing the same solution composition. Type T3 utilized a constant current density of 50 mA/cm^2^ for 1 h, with solutions comprising different concentrations: (1) 0.5 mol/L H_2_SO_4_ + 1 g/L H_2_NCSNH_2_, (2) 0.5 mol/L H_2_SO_4_ + 5 g/L H_2_NCSNH_2_, and (3) 0.5 mol/L H_2_SO_4_ + 10 g/L H_2_NCSNH_2_. The hydrogen charging process resulted in the samples shown in Figure 4, where it was evident that the sample surfaces exhibited darkening effects proportional to the amount of hydrogen charged. After the completion of hydrogen charging, the mechanical behaviors of the material were assessed through tensile testing at a constant displacement rate of 3 mm/min. The experimental equipment was the Sansi UT5105 electronic universal testing machine (Sansi, Shanghai, China), with a maximum tensile force of 100 kN and a testing accuracy of 0.5%. The fracture samples resulting from the different hydrogen charging parameters are shown in Figure 5.

## 3. Results and Discussion

**Figure 6:**
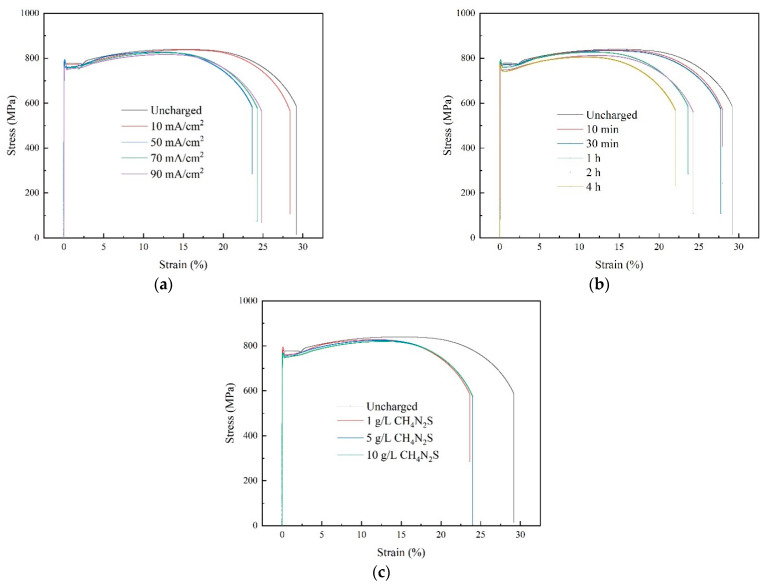
Stress-strain curves of Q690 steels after hydrogen charging: (**a**) current densities, (**b**) times, and (**c**) solution concentrations.

The stress-strain curves of the Q690 high-strength steel specimens obtained through uniaxial tensile testing following various hydrogen charging parameters are shown in Figure 6.

Figure 6a–c illustrate the impacts of the hydrogen charging current density, time, and solution concentration, respectively, on the stress-strain relationship of the material. The experimental findings reveal that the stress-strain curves resulting from different hydrogen charging parameters exhibit four distinct stages: elastic, yield, strain hardening, and necking. Notably, the elastic mechanical properties of the material exhibit minimal changes after electrochemical hydrogen charging. However, the strength of the material decreases with increasing hydrogen charging current density, time, and solution concentration.

The mechanical properties (i.e., tensile strength $\sigma_{b}$, yield stress $\sigma_{s}$, and elongation after fracture $\delta =\Delta L/L_{0}$, where $L_{0}$ and ΔL are the gauge length and gauge length deformation, respectively) resulting from various hydrogen charging parameters are presented in Table 4, Table 5 and Table 6.

An analysis of these tables reveals a consistent trend: the tensile strength, yield stress, and elongation after fracture of the Q690 steel decrease with increasing hydrogen charging current density, time, and solution concentration, respectively. Additionally, the variations in these parameters under uncharged conditions have been calculated and are provided in Table 4, Table 5 and Table 6, where the differences in tensile strength, yield stress, and elongation after fracture are denoted as $\Delta \sigma_{b}=(839.59-\sigma_{b})/839.59,$ $\Delta \sigma_{s}=(772.63-\sigma_{s})/772.63$ and Δδ=(30.4−δ)/30.4, respectively.

The results in Table 4 indicate that under hydrogen charging conditions of a current density of 90 mA/cm^2^, a charging time of 1 h, and a solution concentration of 0.5 mol/L H_2_SO_4_ + 1 g/L H_2_NCSNH_2_, the tensile strength, yield stress, and elongation after fracture decreased by 2.68%, 3.13%, and 20.72%, respectively. Similarly, Table 5 shows that under hydrogen charging conditions of a current density of 50 mA/cm^2^, a charging time of 4 h, and a solution concentration of 0.5 mol/L H_2_SO_4_ + 1 g/L H_2_NCSNH_2_, the tensile strength, yield stress, and elongation after fracture decreased by 4.04%, 4.04%, and 19.47%, respectively. Finally, Table 6 shows that at a current density of 50 mA/cm^2^, a charging time of 1 h, and a solution concentration of 0.5 mol/L H_2_SO_4_ + 10 g/L H_2_NCSNH_2_, the tensile strength, yield stress, and elongation after fracture decreased by 2.29%, 3.33%, and 20.39%, respectively. In summary, the greatest reduction in elongation after fracture is observed under the same hydrogen charging conditions, followed by a decrease in yield stress, with tensile strength exhibiting the smallest reduction.

Using the experimental data from Table 4, Table 5 and Table 6, the mechanical properties (i.e., tensile strength $\sigma_{b}$, yield stress $\sigma_{s}$ and elongation after fracture δ) resulting from various hydrogen charging parameters were analyzed to derive functional relationships. The results presented in Table 7 demonstrate that the mechanical properties exhibit linear correlations with the hydrogen charging current density i, exponential decreases with increasing hydrogen charging time t, and a power law relationship with the hydrogen charging solution concentration c.

**Figure 7:**
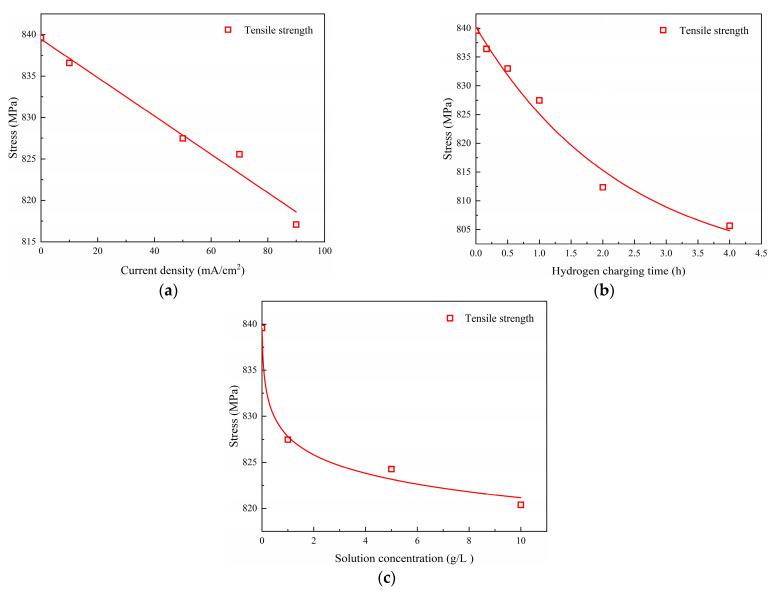
Tensile strength of Q690 steels after hydrogen charging: (**a**) current densities, (**b**) times, and (**c**) solution concentrations.

**Figure 8:**
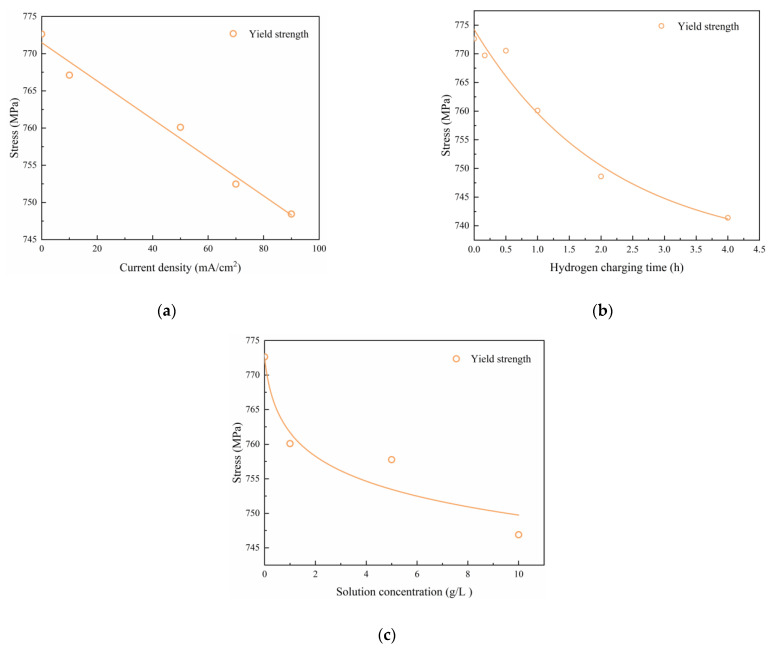
Yield stress of Q690 steels after hydrogen charging: (**a**) current densities, (**b**) times, and (**c**) solution concentrations.

**Figure 9:**
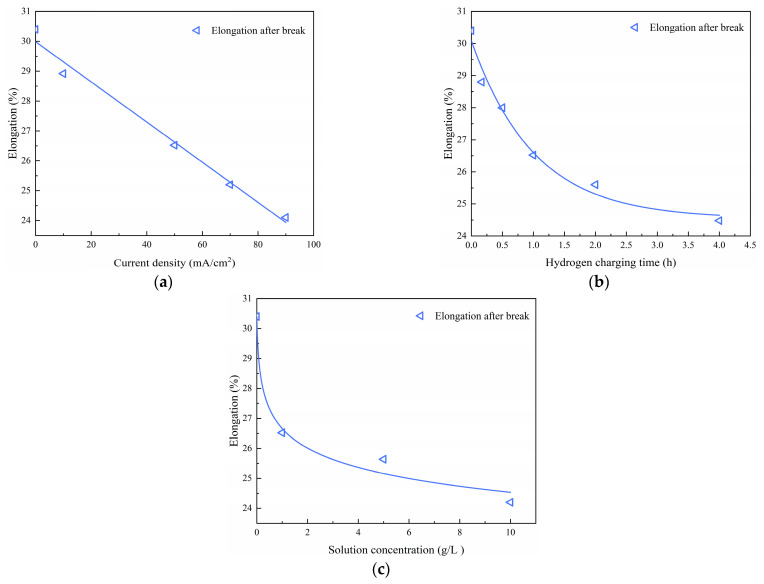
Elongation after fracture of Q690 steels after hydrogen charging: (**a**) current densities, (**b**) times, and (**c**) solution concentrations.

The corresponding fitted curves are shown in Figure 7, Figure 8 and Figure 9.

A comparison between the test values and fitted values for different hydrogen charging parameters is provided in Table 8, indicating that the mean values of the parameters ($\sigma_{b,cal}/\sigma_{b}$, $\sigma_{s,cal}/\sigma_{s}$ and $\delta_{cal}/\delta$) are close to 1.00 and that the corresponding coefficient of variation (COV) is 0.0. Overall, the fitted curves accurately depict the influences of hydrogen charging parameters on the material parameters [28,29,30,31].

To comparatively assess the impacts of hydrogen charging parameters on the mechanical characteristics of the Q690 high-strength steel, the parameters $\sigma_{b}$, $\sigma_{s}$ and δ were normalized. The corresponding reference values $\sigma_{b0}$, $\sigma_{s0},$ and $\delta_{0}$ are 839.59 MPa, 772.63 MPa, and 30.40%, respectively. The normalized results are presented in Table 9.

Additionally, the hydrogen charging current density i, hydrogen charging time t, and hydrogen charging solution concentration c were normalized to the reference values $i_{0}$, $t_{0},$ and $c_{0}$, which have values of 82.26 mA/cm^2^, 1.43 h, and 10 g/L, respectively. The reference values $i_{0}$, $t_{0},$ and $c_{0}$ are calculated with Equations (1)–(3) in Table 7, while $\sigma_{b}$ decreases to 820.39 MPa. ${\overset{–}{\sigma }}_{b,cal}$, ${\overset{–}{\sigma }}_{s,cal}$, and ${\overset{–}{\delta }}_{cal}$ are the normalized parameters of the tensile strength, yield stress, and elongation after fracture, respectively. $\overset{–}{i}$, $\overset{–}{t}$ and $\overset{–}{c}$ are normalized parameters for i, t, and c, respectively. The normalized equations for the mechanical properties are shown in Table 10 and are similar to those shown in Table 7.

**Figure 10:**
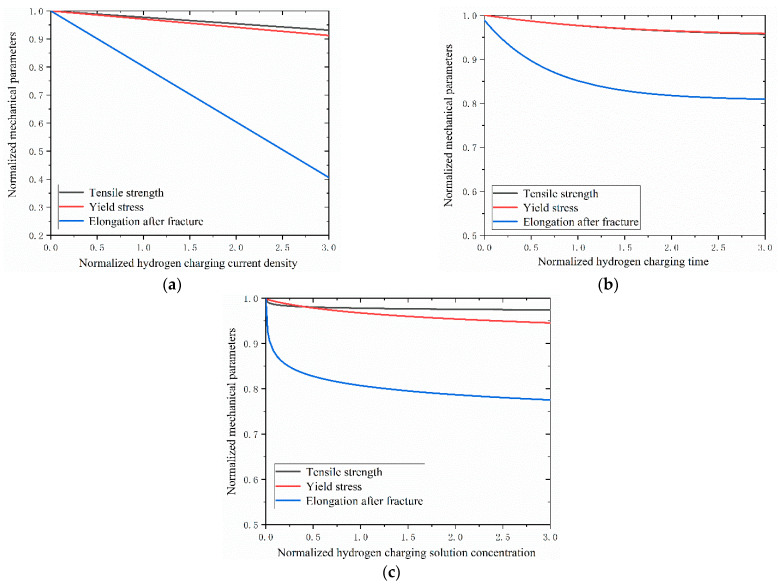
Mechanical properties of Q690 steel with hydrogen charging parameters: (**a**) current densities, (**b**) times, and (**c**) solution concentrations.

**Figure 11:**
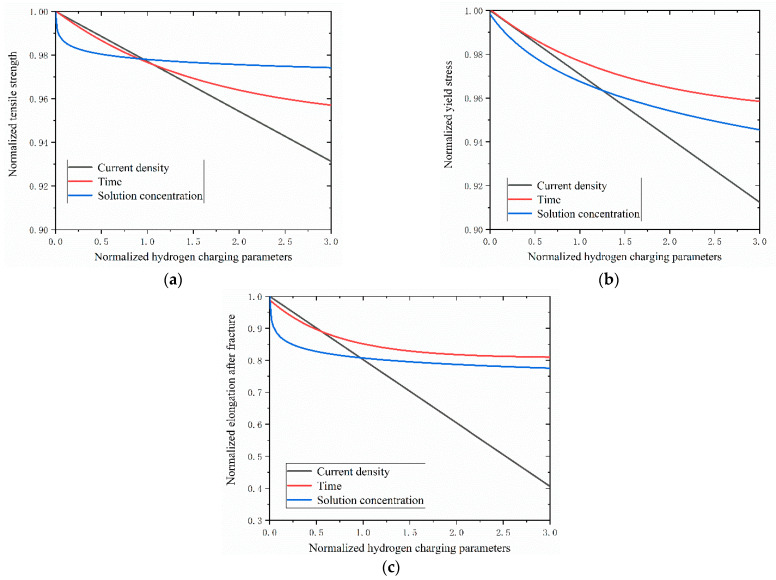
Mechanical properties of Q690 steels after hydrogen charging: (**a**) tensile strength, (**b**) yield stress, and (**c**) elongation after fracture.

The impacts of hydrogen charging parameters on the material properties of the Q690 high-strength steel are shown in Figure 10 and Figure 11.

The results reveal decreases in the mechanical properties of the Q690 steel specimens with increasing hydrogen charging current density, time, and solution concentration (Figure 10). Notably, the most significant effects of hydrogen charging parameters on the mechanical properties are observed for the elongation after fracture, followed by the yield stress, with tensile strength exhibiting the least degradation (Figure 10). Furthermore, the degradation curves of tensile strength and yield stress mirror those of the hydrogen charging times (Figure 10b). Additionally, the results indicate the presence of a linear relationship between the mechanical behavior and the hydrogen charging current density, while the relationship with the hydrogen charging time/solution concentration is nonlinear (Figure 11). For the tensile strength, when the normalized hydrogen charging parameter is less than 1.0, the hydrogen charging solution concentration exerts the most significant influence on the material performance, followed by the hydrogen charging time and the hydrogen charging current density. Conversely, when the normalized hydrogen charging parameter exceeds 1.0, the effect of the hydrogen charging parameter on the material performance is reversed (Figure 11a). Regarding the yield stress, the impacts of the hydrogen charging current densities and times on the material parameters are nearly identical when the normalized hydrogen charging parameter is less than 0.5. Subsequently, the influence of the hydrogen charging time gradually diminishes. When the normalized hydrogen charging parameter falls below 1.5, the effects of the hydrogen charging solution concentration on the material properties outweigh those of the hydrogen charging current density and hydrogen charging time. Beyond this threshold, the effects of the hydrogen solution concentration on the material properties are between those of the hydrogen charging time and the current density (Figure 11b). For the yield stress, when the normalized hydrogen charging parameter is less than 0.6, the hydrogen charging solution concentration has the most pronounced effect on the material properties, followed by the hydrogen charging time and the hydrogen charging current density. Between 0.6 and 1.0, the hydrogen charging solution concentration remains the primary influencing factor, followed by the hydrogen charging current density and the hydrogen charging time. Above 1.0, the hydrogen charging parameters exert the greatest impacts on the material properties, followed by the hydrogen current density, hydrogen charging solution concentration, and hydrogen charging time (Figure 11c).

**Figure 12:**
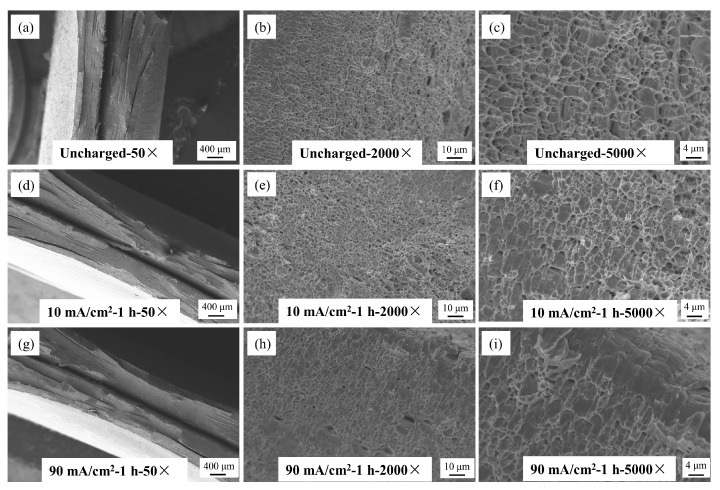
SEM map of fracture morphology of Q690 high-strength steel welded parts under different hydrogen charging current densities. (**a**) Unchanged 50×; (**b**) Unchanged 2000×; (**c**) Unchanged 5000×; (**d**) 10 mA/cm^2^-1 h 50×; (**e**) 10 mA/cm^2^-1 h 2000×; (**f**) 10 mA/cm^2^-1 h 5000×; (**g**) 50 mA/cm^2^-1 h 50×; (**h**) 50 mA/cm^2^-1 h 2000×; (**i**) 50 mA/cm^2^-1 h 5000×.

**Figure 13:**
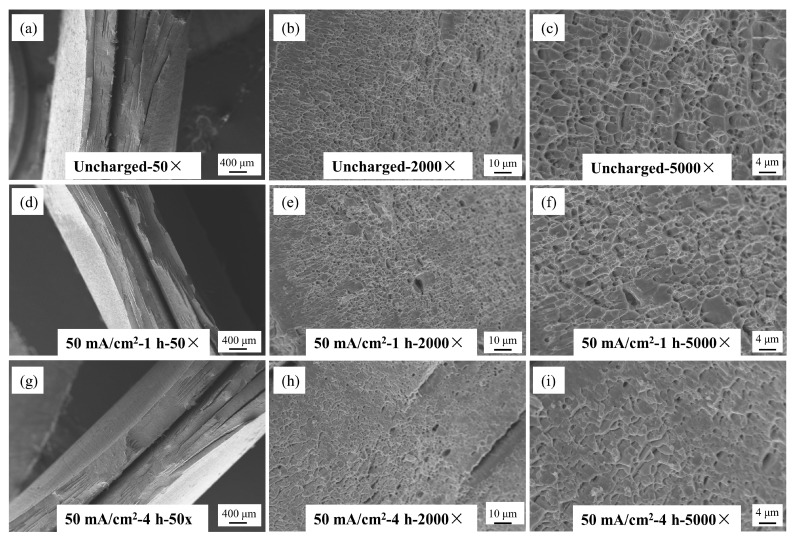
SEM map of fracture morphology of Q690 high-strength steel welded parts under different hydrogen charging times. (**a**) Unchanged 50×; (**b**) Unchanged 2000×; (**c**) Unchanged 5000×; (**d**) 50 mA/cm^2^-1 h 50×; (**e**) 50 mA/cm^2^-1 h 2000×; (**f**) 50 mA/cm^2^-1 h 5000×; (**g**) 50 mA/cm^2^-4 h 50×; (**h**) 50 mA/cm^2^-4 h 2000×; (**i**) 50 mA/cm^2^-4 h 5000×.

**Figure 14:**
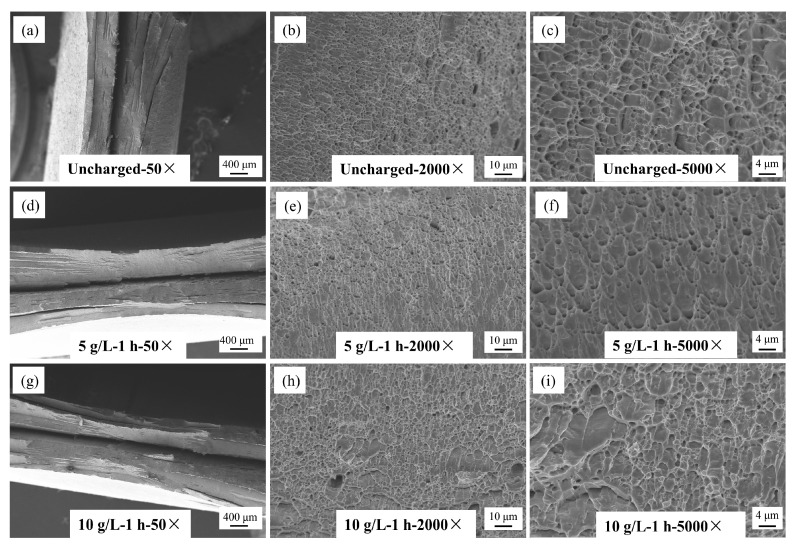
SEM map of fracture morphology of Q690 high-strength steel welded parts under different hydrogen solution concentrations. (**a**) Unchanged 50×; (**b**) Unchanged 2000×; (**c**) Unchanged 5000×; (**d**) 5 g/L-1 h 50×; (**e**) 5 g/L-1 h 2000×; (**f**) 5 g/L-1 h 5000×; (**g**) 10 g/L-1 h 50×; (**h**) 10 g/L-1 h 2000×; (**i**) 10 g/L-1 h 5000×.

The fracture morphology of Q690 high-strength steel under various hydrogen charging conditions is illustrated in Figure 12, Figure 13 and Figure 14. These images, captured using a Zeiss EVO18 scanning electron microscope (SEM), Zeiss, Oberkochen, Germany, at magnifications of 50×, 2000×, and 5000×, reveal detailed insights into the material’s behavior. Figure 12 illustrates the macro- and microstructural changes in the fracture morphology of the material under varying hydrogen charging current densities (10 mA/cm^2^ and 90 mA/cm^2^). On a macro scale, the fracture surface of the uncharged sample displays pronounced necking with a rough, uneven texture. As the hydrogen charging current density increases, necking remains observable but becomes less severe, and the fracture surface progressively smooths. At the microstructural level, the uncharged sample shows a typical ductile dimpled morphology, which is characteristic of ductile fracture. With higher hydrogen charging current densities, the number of dimples markedly decreases, with some areas nearly devoid of dimples, indicating a transition to a brittle fracture mode. This shift suggests a gradual change in the fracture mechanism towards increased brittleness. Figure 13 illustrates the effects of hydrogen charging time (1 h and 4 h) on fracture morphology and mode, revealing trends analogous to those observed with varying hydrogen charging current densities. In the uncharged state, the macro-fracture displays pronounced necking. As hydrogen charging time increases, necking becomes less prominent, although not completely eliminated, and the fracture surface smooths out. At the microstructural level, the typical dimpled morphology present before hydrogen charging undergoes significant alteration after 4 h of charging, with a substantial decrease in the number of dimples. These changes collectively suggest a transition in fracture mode from ductile to brittle. Figure 14 further explores the impact of varying solution concentrations (5 g/L and 10 g/L) on fracture morphology, with results aligning with the trends observed for hydrogen-charging current density and duration. In the uncharged condition, the macroscopic fracture exhibits significant necking. As solution concentration increases, necking diminishes, and the fracture surface becomes smoother. Microscopic observations reveal that the typical dimpled morphology present before hydrogen charging undergoes a marked transformation with increased solution concentration and hydrogen treatment, resulting in a reduced number of dimples. These observations collectively suggest a shift towards a more brittle fracture mode [32]. In summary, under uncharged conditions, the macroscopic fracture displays significant necking. As hydrogen-charging parameters increase, macroscopic necking gradually disappears, the number of microscopic dimples decreases, and the material ultimately transitions to a fully brittle fracture. This shift occurs because as the quantity of hydrogen atoms penetrating the material increases, they diffuse more freely within the metal matrix and eventually accumulate as hydrogen molecules at microscopic voids and defects, driving the transition from ductile to brittle fracture.

## 4. Conclusions

In this study, the mechanical behaviors of Q690 high-strength structural steel were investigated after performing various hydrogen charging parameters. Specific parameters, including the tensile strength, yield stress, and elongation after fracture, were analyzed to assess the influences of the hydrogen charging current density, time, and solution concentration. The findings are summarized as follows:

1. The stress-strain relationship of Q690 steel maintained the characteristic phases of elasticity, yielding, strengthening, and necking after electrochemical hydrogen charging. Notably, the elastic mechanical properties of this steel exhibited minimal alteration after electrochemical hydrogen charging. However, significant changes in the mechanical properties were observed after the elastic phase due to electrochemical hydrogen charging.
2. The mechanical properties (tensile strength, yield stress, and elongation after fracture) following electrochemical hydrogen charging were expressed as functions of the hydrogen charging parameters. Specifically, these parameters demonstrated linear relationships with the hydrogen charging current density, exponential decreases with the hydrogen charging time, and power law relationships with the hydrogen charging solution concentration.
3. To comparatively assess the impacts of hydrogen charging parameters on the mechanical behaviors of the high-strength Q690 steel specimens, the mechanical properties and hydrogen charging parameters were normalized. Based on this normalization, the effects of the hydrogen charging current density, time, and solution concentration on the mechanical properties were individually investigated. Additionally, the influences of the normalized electrochemical parameters on the tensile strength, yield stress, and elongation after fracture were analyzed.
4. The macroscopic fracture displays significant necking in uncharged conditions. As hydrogen-charging parameters increase, macroscopic necking gradually disappears, the number of microscopic dimples decreases, and the material ultimately transitions to a fully brittle fracture.

## Figures and Tables

**Table 1:** Main chemical composition (mass fraction %).

Chem. Comp.	C	Mn	Si	P	S	Cr	Ni	Mo	Nb	V	Ti	B
Q690	0.17	1.38	0.41	0.22	0.18	1.12	1.38	0.54	0.10	0.11	0.18	0.0034

**Table 2:** Mechanical behaviors of the Q690 steel specimens.

Mat.	E (GPa)	$\sigma_{S}$ (MPa)	$\sigma_{b}$ (MPa)	δ (%)
Q690	210.28	839.59	772.63	30.40

E: elastic modulus; $\sigma_{S}$: yield stress; $\sigma_{b}$: tensile strength; δ: elongation after fracture.

**Table 3:** Hydrogen charging parameters.

Type	Current Density (mA/cm^2^)	Time (h)	Solution Concentrations (g/L)
T1	0, 10, 50, 70, 90	1	1
T2	50	0, 1/6, 1/2, 1, 2,4	1
T3	50	1	0, 1, 5, 10

**Table 4:** Mechanical properties of the Q690 steels with different hydrogen charging current densities.

Current Density	$\sigma_{b}$ (MPa)	$\sigma_{S}$ (MPa)	δ (%)	${∆\sigma }_{b}$ (%)	${∆\sigma }_{S}$ (%)	∆δ (%)
Uncharged	839.59	772.63	30.40	0	0	0
10 mA/cm^2^	836.58	767.11	28.92	0.36	0.71	4.87
50 mA/cm^2^	827.48	760.10	26.52	1.44	1.62	12.76
70 mA/cm^2^	825.56	752.46	25.20	1.67	2.61	17.11
90 mA/cm^2^	817.08	748.44	24.10	2.68	3.13	20.72

**Table 5:** Mechanical properties of high-strength steels with different hydrogen charging times.

Time	$\sigma_{b}$ (MPa)	$\sigma_{S}$ (MPa)	δ (%)	${∆\sigma }_{b}$ (%)	${∆\sigma }_{S}$ (%)	∆δ (%)
Uncharged	839.59	772.63	30.40	0	0	0
10 min	836.43	769.71	28.80	0.38	0.38	5.26
30 min	833.00	770.54	28.00	0.78	0.27	7.89
1 h	827.48	760.10	26.52	1.44	1.62	12.76
2 h	812.35	748.61	25.60	3.24	3.11	15.79
4 h	805.66	741.40	24.48	4.04	4.04	19.47

**Table 6:** Mechanical properties of Q690 steels at various hydrogen charging solution concentrations.

Solution Concentration	$\sigma_{b}$ (MPa)	$\sigma_{S}$ (MPa)	δ (%)	${∆\sigma }_{b}$ (%)	${∆\sigma }_{S}$ (%)	∆δ (%)
Uncharged	839.59	772.63	30.40	0	0	0
1 g/L	827.48	760.10	26.52	1.44	1.62	12.76
5 g/L	824.27	757.77	25.64	1.82	1.92	15.66
10 g/L	820.39	746.90	24.20	2.29	3.33	20.39

**Table 7:** Functions of the mechanical properties of the Q690 steels after hydrogen charging.

Parameters	Conditions	Equations	
$\sigma_{b,cal}$	Current densities	$\sigma_{b,cal}=839.59-0.2334\cdot i$	(1)
	Times	$\sigma_{b,cal}=797.14+43.08\cdot \exp(-t/2.32)$	(2)
	Solution concentrations	$\sigma_{b,cal}=827.88\cdot {\left(c+0.0189\right)}^{-0.00354}$	(3)
$\sigma_{s,cal}$	Current densities	$\sigma_{s,cal}=772.63-0.2738\cdot i$	(4)
	Times	$\sigma_{s,cal}=735.28+37.51\cdot \exp(-t/2.13)$	(5)
	Solution concentrations	$\sigma_{s,cal}=762.58\cdot {\left(c+0.171\right)}^{-0.00731}$	(6)
$\delta_{cal}$	Current densities	$\delta_{cal}=30.40-0.0731\cdot i$	(7)
	Times	$\delta_{cal}=24.54+5.54\cdot \exp(-t/1.01)$	(8)
	Solution concentrations	$\delta_{cal}=26.69\cdot {\left(c+0.0282\right)}^{-0.0365}$	(9)

**Table 8:** Comparison of test values and fitted values for various hydrogen charging parameters.

Methods	Charging Parameters	Fitted Value/Test Value		
		$\sigma_{b,cal}/\sigma_{b}$	$\sigma_{s,cal}/\sigma_{s}$	$\delta_{cal}/\delta$
**T1**	Uncharged	1.00	1.00	1.00
	10 mA/cm^2^	1.00	1.00	1.03
	50 mA/cm^2^	1.00	1.00	1.01
	70 mA/cm^2^	1.00	1.00	1.00
	90 mA/cm^2^	1.00	1.00	0.99
T2	Uncharged	1.00	1.00	0.99
	10 min	1.00	1.00	1.02
	30 min	1.00	0.99	1.00
	1 h	1.00	1.00	1.00
	2 h	1.00	1.00	0.99
	4 h	1.00	1.00	1.01
T3	Uncharged	1.00	1.00	1.00
	1 g/L	1.00	1.00	1.01
	5 g/L	1.00	0.99	0.98
	10 g/L	1.00	1.00	1.01
	Mean	1.00	1.00	1.00
	COV	0.00	0.00	0.00

**Table 9:** Normalized mechanical properties of Q690 steels subjected to various hydrogen charging parameters.

Methods	Charging Parameters	Test Value/Reference Value		
		$\sigma_{b,cal}/\sigma_{b}$	$\sigma_{s,cal}/\sigma_{s}$	$\delta_{cal}/\delta$
**T1**	Uncharged	1.00	1.00	1.00
	10 mA/cm^2^	0.996	0.993	0.951
	50 mA/cm^2^	0.986	0.984	0.872
	70 mA/cm^2^	0.983	0.974	0.829
	90 mA/cm^2^	0.973	0.969	0.793
T2	Uncharged	1.00	1.00	1.00
	10 min	0.996	0.996	0.947
	30 min	0.992	0.997	0.921
	1 h	0.986	0.984	0.872
	2 h	0.968	0.969	0.842
	4 h	0.960	0.960	0.805
T3	Uncharged	1.00	1.00	1.00
	1 g/L	0.986	0.984	0.872
	5 g/L	0.982	0.981	0.843
	10 g/L	0.977	0.967	0.796

**Table 10:** Functions of the normalized parameters of the Q690 steels after hydrogen charging.

Parameters	Conditions	Equations	
${\overset{–}{\sigma }}_{b,cal}$	Current densities	${\overset{–}{\sigma }}_{b,cal}=1-0.0229\cdot \overset{–}{i}$	(10)
	Times	${\overset{–}{\sigma }}_{b,cal}=0.949+0.0513\cdot \exp(-\overset{–}{t}/1.619)$	(11)
	Solution concentrations	${\overset{–}{\sigma }}_{b,cal}=0.978\cdot {\left(\overset{–}{c}+0.00189\right)}^{-0.00354}$	(12)
${\overset{–}{\sigma }}_{s,cal}$	Current densities	${\overset{–}{\sigma }}_{s,cal}=1-0.0292\cdot \overset{–}{i}$	(13)
	Times	${\overset{–}{\sigma }}_{s,cal}=0.971+0.0485\cdot \exp(-\overset{–}{t}/1.490)$	(14)
	Solution concentrations	${\overset{–}{\sigma }}_{s,cal}=0.971\cdot {\left(\overset{–}{c}+0.0171\right)}^{-0.00731}$	(15)
${\overset{–}{\delta }}_{cal}$	Current densities	${\overset{–}{\delta }}_{cal}=1-0.198\cdot \overset{–}{i}$	(16)
	Times	${\overset{–}{\delta }}_{cal}=0.807+0.182\cdot \exp(-\overset{–}{t}/0.706)$	(17)
	Solution concentrations	${\overset{–}{\delta }}_{cal}=0.807\cdot {\left(\overset{–}{c}+0.00282\right)}^{-0.0365}$	(18)

## Data Availability

Data are contained within the article.
